# Efficacy and safety of pregabalin 600 mg/d for treating painful diabetic peripheral neuropathy: A double-blind placebo-controlled trial

**DOI:** 10.1186/1471-2377-8-33

**Published:** 2008-09-16

**Authors:** Joseph C Arezzo, Julio Rosenstock, Linda LaMoreaux, Lynne Pauer

**Affiliations:** 1 Albert Einstein College of Medicine, New York, NY, USA; 2 Dallas Diabetes and Endocrine Center, Dallas, TX, USA; 3 Pfizer Global Research & Development, Ann Arbor, MI, USA

## Abstract

**Background:**

Recent consensus guidelines recommend pregabalin as a first-tier treatment for painful diabetic peripheral neuropathy (DPN). We evaluated the efficacy of pregabalin 600 mg/d (300 mg dosed BID) versus placebo for relieving DPN-associated neuropathic pain, and assessed its safety using objective measures of nerve conduction (NC).

**Methods:**

In this randomized, double-blind, placebo-controlled trial, the primary efficacy measure was endpoint mean pain score (MPS) from daily pain diaries (11-point scale). NC velocity and sensory and motor amplitudes were assessed at baseline, endpoint, and end of follow-up (2 weeks post-treatment). At each timepoint, the median-motor, median-sensory, ulnar-sensory, and peroneal-motor nerves were evaluated. Secondary efficacy measures included weekly MPS and proportion of responders (patients achieving ≥50% reduction in MPS from baseline to endpoint). After 1-weeks' dosage escalation, pregabalin-treated patients received 300 mg BID for 12 weeks.

**Results:**

Eighty-two patients received pregabalin and 85 placebo. Mean durations were 10 years for diabetes and ~5 years for painful DPN. Pregabalin-treated patients had lower MPS than controls (mean difference, -1.28; p <.001). For all four nerves, 95% CIs for median differences in amplitude and velocity from baseline to endpoint and baseline to follow-up included 0 (ie, no significant difference vs. placebo). Significant pain improvement among pregabalin-treated patients was evident at week 1 and sustained at every weekly timepoint. More pregabalin-treated patients (49%) than controls (23%) were responders (p <.001).

**Conclusion:**

Pregabalin 600 mg/d (300 mg BID) effectively reduced pain, was well tolerated, and had no statistically significant or clinically meaningful effect on NC in patients with painful DPN.

**Trial registration:**

ClinicalTrials.gov NCT00159679

## Background

Diabetic peripheral neuropathy (DPN) occurs in approximately 20% of all diabetics [1], and among persons who have had diabetes >25 years, its prevalence is about 50%. Pathologic features of DPN include distal axonopathy, primary demyelination, axoglial disjunction, and Wallerian degeneration with a consequent loss of fiber density [2,3]. Typical symptoms of DPN include tingling, pain, numbness or weakness in the feet and hands; severe dysesthetic burning with nighttime worsening; allodynia; insomnia; and anxiety or depression [4-7].

Pregabalin is a nonopiate that is well tolerated and relieves painful symptoms of distal symmetrical polyneuropathy with minimal risk of dependence or impact on patients' diabetes control [8]. Pregabalin has consistently proved an effective treatment for DPN and postherpetic neuralgia (PHN) in its extensive clinical trial program [9-16]. It is among the agents recommended by the American Academy of Neurology as a Group 1 treatment for PHN [17], and as a first-line treatment for painful polyneuropathy by the European Federation of Neurological Societies [18]. Recent consensus guidelines have identified pregabalin as one of the first-tier treatments for painful DPN [19,20].

The current study evaluated the efficacy and safety/tolerability of pregabalin at the upper end of its recommended dosing range (600 mg/d) and at a simpler regimen, twice daily. The study also explored the impact of pregabalin on sensory and motor nerve conduction (NC). In a recent study, monotone worsening of velocity was demonstrated to be sensitive to even subclinical progression of neuropathy associated with diabetes [21]. The electrophysiologic measures employed in this study were a standard, validated, objective, and reliable measure of the functional integrity of large diameter peripheral nerve fibers [22-24].

## Methods

This was a 13-week, randomized, double-blind, placebo-controlled, parallel-group trial performed across 23 centers in the United States. Following a 1-week baseline phase (visit 1), patients were randomized (visit 2/visit 3, week 0 titration initiation) to placebo or to pregabalin 600 mg/d administered as two divided doses. Daily dosage was escalated over a 1-week period beginning with a single dose of 150 mg pregabalin on day 1, followed by two doses of 150 mg pregabalin on days 2–6 and two doses of 300 mg pregabalin on day 7 (end of titration, visit 4) which were continued for 12 weeks (visits 4–7). No dosage changes were allowed during the study. Following study endpoint (termination visit 8, week 13), at which time pregabalin was discontinued without taper, there was a 2-week follow-up period (visits 9 and 10), during which no drug was administered. Patients who withdrew from treatment early were required to complete all termination and follow-up visit procedures.

Patients were randomly assigned to treatment according to a computer-generated random code, which was prepared by the sponsor. The sponsor distributed the number-coded study medications to the study sites, which were assigned using an interactive voice-response system. Study sites also received dispensing instructions and forms to document medication usage from the sponsor. The sponsor, members of the study site, and the patients were unaware of the treatment assignment. Blinding was maintained by dispensing pregabalin and placebo in identical capsules.

The final protocol and informed consent documentation were reviewed and approved by the Institutional Review Board(s) (IRB) and/or Independent Ethics Committee(s) (IEC) at each of the investigational centers participating in the study. This study was conducted in compliance with the ethical principles according to the Declaration of Helsinki (revised Edinburgh, 2000) and in compliance with IRB/IEC, informed consent regulations, and International Congress of Harmonization (ICH) Good Clinical Practices (GCP). Written informed consent was obtained prior to the subject entering the study (before initiation of protocol-specified procedures).

### Key inclusion criteria

Enrolled patients were men or women of any race ≥18 years of age. Patients must have had type 1 or type 2 diabetes with HbA_1C _≤11% and, if on antidiabetic medication, must have been on a stable antidiabetic medication regimen for 30 days prior to randomization. Duration of painful DPN was required to be ≥3 months, and patients must have scored ≥40 mm on the Short-Form McGill Pain Questionnaire (SF-MPQ) Visual Analog Scale. (VAS) [25]. Patients were required to keep a daily pain diary, in which they recorded their daily pain score on an 11-point (0 = "no pain" to 10 = "worst possible pain") numeric rating scale (NRS) [26,27]. At randomization, patients must have completed at least four daily pain entries and had an average daily pain score ≥4 over the past 7 days.

### Key exclusion criteria

Patients with creatinine clearance rates (CL_cr_) of ≤60 mL/min were excluded, as were patients with any conditions that could confound assessment of pain due to DPN. Prior use of potential retinotoxins (including hydroxychloroquine, deferoxamine, thioridazine, and vigabatrin) was a cause for exclusion, as was the use of the following prohibited medications without appropriate washout:

▪ Medications and supplements commonly used for relief of neuropathic pain (≥7 days prior to visit 3)

▪ Antiepileptics (≥7 days prior to visit 3)

▪ Antidepressants (≥7 days prior to visit 3; except for stable [>30 days] regimens of SSRIs for treatment of anxiety or depression)

▪ NSAIDs (including COX-2 inhibitors), dextromethorphan (≥1 day prior to visit 3)

Aspirin (up to 325 mg/d for cardiac and stroke prophylaxis), acetaminophen (up to 4 g/d), SSRIs (see above), and benzodiazepines such as lorazepam (dosed at bedtime with stable [>30 days] regimen for sleep problems) were allowed.

### Primary efficacy parameter

The primary efficacy instrument was the 11-point NRS in patients' daily pain diaries. Each day on awakening, patients described their pain during the previous 24 hours by choosing the appropriate number from 0–10. The primary analysis compared endpoint mean pain score (calculated as the mean of the last seven available diary entries while on double-blind study drug, including diary entry on the day after the last dose) between pregabalin and placebo groups. Analysis of covariance (ANCOVA) was used with treatment and center in the model and baseline mean pain score (mean of the last seven diary entries prior to taking study drug, including diary entry on day 1) as covariate. The definition of endpoint mean pain constituted a last observation carried forward (LOCF) approach for patients who did not complete the study. Analysis using baseline observation carried forward (BOCF) for noncompleters was performed as a sensitivity analysis.

### Supplemental analyses

Weekly mean pain scores were evaluated using ANCOVA, with the same main effects model as for endpoint, as a supplemental analysis. Proportion of responders – those patients with ≥50% reduction in mean pain score from baseline to endpoint – was compared between pregabalin and placebo groups using the Cochran-Mantel-Haenszel procedure, adjusting for center.

### Secondary efficacy parameters

Patients evaluated pregabalin's effect on pain-associated sleep interference using an 11-point NRS (0 = "pain did not interfere with sleep" to 10 = "pain completely interfered with sleep") recorded in daily sleep diaries. Analyses of endpoint and weekly sleep-interference scores were performed as described above for mean pain scores.

As an additional measure of patients' pain and its characteristics, the SF-MPQ was administered at screening, randomization, and weeks 1, 5, 9, and 13. The questionnaire includes 15 descriptors, each ranked by patients on a 4-point intensity scale, as well as the 100-mm VAS and the 6-point Present Pain Intensity (PPI) index. Sensory, affective, and total scores for pain descriptors; VAS score; and PPI were analyzed separately at endpoint and by visit using ANCOVA with treatment and center in the model and using the scores at randomization as the covariate.

To gauge global improvement, the Clinical and Patient Global Impression of Change scales (CGIC and PGIC) [28] were administered at endpoint. Both scales were analyzed using modified ridit transformation with the Cochran-Mantel-Haenszel procedure, adjusting for center.

### Primary safety parameter

The primary safety parameter was nerve conduction (NC), assessed at three timepoints during the study – baseline, endpoint, and follow-up. At each timepoint, replicate measures (at least 1 calendar day apart [maximum of 7 calendar days apart]) were taken to reduce variability. Mean NC measures were compared at baseline and endpoint to determine whether there was any change in NC within large, myelinated nerve fibers during the study. NC testing was performed during follow-up to determine whether any NC deficits that may have been observed at endpoint would be reversible after 2 weeks off study medication. All patients were to complete the follow-up NC testing, regardless of whether they completed the study. No imputation was done for NC values that were missing as a result of technical error, were not performed, or performed outside of allowable time frames. For measurements recorded as physiologically absent ("no response," meaning outside detection sensitivity of the test), non-zero values were imputed using the 1st percentile of all non-missing values across all visits, all patients, and all treatment groups. This was done to impute the low end of the range of detectable values while avoiding undue influence by outliers.

Amplitude and conduction velocity were assessed for the peroneal motor nerve, median motor nerve, median sensory nerve, and ulnar sensory nerve using standard surface recording procedures [29]. Amplitude was measured from baseline-to-peak in millivolts (mV) for the muscles innervated by the motor nerves and in microvolts (μV) for direct measure of sensory nerves. Conduction velocity was measured in meters per second (m/s) and was recorded at the onset of response. NC data were recorded on patients' left sides (unless contraindicated). Skin temperature was to be maintained at ≥32.0°C (89.6°F) in the arm and at ≥31.0°C (87.8°F) in the leg throughout testing; if necessary, warming techniques were allowed to achieve minimum skin temperatures. Surface temperatures were taken several minutes after warming to allow stabilization [30]. Presence of peripheral edema in the limb was to be noted at the time of NC testing.

### Analysis

Change from baseline to endpoint and from baseline to follow-up was computed for each NC parameter. Differences in medians between treatment groups and the corresponding 95% confidence intervals (CIs) were provided; the CIs were computed using Campbell's and Gardner's algorithm [31]. If the CIs at endpoint included 0, this suggested the effect of pregabalin on NC parameters was no different from that of placebo. For each parameter, patients with possible outlying changes (any value ≤ the 5th percentile) were identified for each treatment group at endpoint and follow-up, and these changes were reviewed for clinical relevance.

### Additional safety measures

In addition to NC parameters, safety and tolerability were evaluated by collection of all observed or volunteered adverse events (AEs). Severity was assessed as mild, moderate, or severe, and investigators' opinions of the relation of observed or volunteered AEs to treatment were recorded. Serious AEs were those that resulted in death, were life-threatening, required inpatient hospitalization or prolongation of existing hospitalization, resulted in a persistent or significant disability/incapacity, or resulted in congenital anomaly/birth defect.

### AEs of special interest

Detailed assessments of edema were regularly made, at baseline and weeks 1, 5, 9, and 13. Peripheral edema was quantified as absent, trace, pitting +1, pitting +2, pitting +3, and patients were assessed for the presence or absence of facial and generalized edema. Additionally, a simpler edema assessment (absent or present in the limb tested) was performed in conjunction with NC testing, so edema was assessed (either in detail or simply) at every study visit. Weight change was assessed in two ways: spontaneous reports of weight change as an AE by patients and objective measure of weight change ≥7% from baseline to endpoint.

Laboratory evaluations included hematology, chemistry, urinalysis, CL_cr_, thyroid-stimulating hormone, glycosylated hemoglobin (HbA_1c_), and serum pregnancy testing. Neurologic and physical examinations were performed. Clinically significant changes in physical examination findings and abnormal laboratory findings were noted.

## Results

### Patient disposition

**Figure 1:**
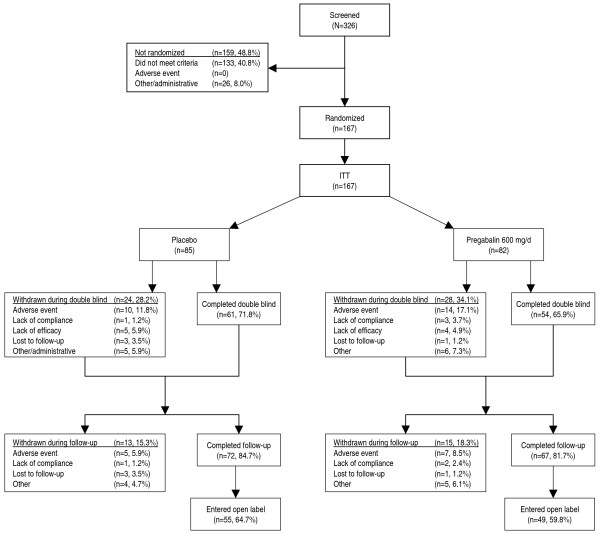
Patient disposition.

Of 326 patients who entered baseline, 167 were randomized and received study medication (representing the intent-to-treat [ITT] population, Figure 1). The most frequent reason patients were not randomized was failure to meet entry criteria (133/159). One hundred fifteen patients completed double-blind treatment, and 139 completed follow-up. The percentages of discontinuations during the double-blind phase were similar: pregabalin (34%) and placebo (28%). Most of these withdrawals were due to AEs, with 17% discontinuing pregabalin and 12% discontinuing placebo. During follow-up, AEs were again the most common reason for withdrawal, with 9% discontinuing from the pregabalin and 6% discontinuing from the placebo groups.

Patients in the two treatment groups were well matched for demographic and baseline characteristics (Table 1). Patients' use of prior and concomitant medications for diabetes control were also similar between treatment groups: 81 of 85 patients in the placebo group were taking antidiabetic medications, including metformin/metformin HCl (48%), glipizide (18%), pioglitazone (15%), and insulin (15%), whereas 76 of 82 patients in the pregabalin group were taking antidiabetic medications, including metformin/metformin HCl (50%), glipizide (23%), pioglitazone (22%), insulin (11%), and rosiglitazone/rosiglitazone maleate (13%). Seven patients in the placebo group and 6 in the pregabalin group used concomitant acetaminophen for pain during the study as allowed per protocol.

**Table 1:** Patient characteristics

	Placebo (n = 85)	Pregabalin (n = 82)
Male, n (%)	45 (52.9)	58 (70.7)
Race, n (%)		
White	61 (71.8)	62 (75.6)
Black	12 (14.1)	9 (11.0)
Hispanic	12 (14.1)	9 (11.0)
Other	0	2 (2.4)
Age, years		
Mean (SD)	58.3 (10.9)	58.2 (9.6)
Median	60	59.5
Range (min-max)	32–86	31–79
Body Mass Index, (kg/m^2^)		
Mean (SD)	35.8 (8.4)	36.6 (8.3)
Range (min-max)	21.7–61.9	23.5–60.2
Weight, kg		
Mean (SD)	105 (23)	107 (24)
Median	104	102
Range (min-max)	59–174	61–182
Diabetes type, n (%)		
Type 1	9 (11)	4 (5)
Type 2	76 (89)	78 (95)
Duration of diabetes, years		
Mean (SD)	10.3 (8.6)	10.3 (8.2)
Median	7.5	8.8
Range (min-max)	0.3–42.2	0.6–42.8
Duration of painful DPN, years		
Mean (SD)	4.4 (3.7)	4.9 (3.4)
Median	3.5	4.5
Range (min-max)	0.3–18.4	0.3–14.8
Distribution of pain, n (%)		
Lower extremities	85 (100)	82 (100)
Upper extremities	37 (44)	32 (39)
Baseline mean pain score		
Mean (SD)	6.58 (1.58)	6.28 (1.47)
Median	6.57	6.14
Range (min-max)	3.71–10.00	3.57–9.71

### Primary efficacy parameter

Treatment with 600 mg/d pregabalin (300 mg BID) resulted in improvement, compared with placebo, in endpoint mean pain score: for pregabalin, endpoint mean pain score was 3.54, whereas for placebo, it was 4.82, for a treatment difference = -1.28 (95% confidence interval [CI], -1.96 to -0.60; p = .0003). Using BOCF as a sensitivity analysis, pregabalin was also superior to placebo (endpoint mean scores, 4.32 and 5.03, treatment difference = -0.71; 95% CI, -1.39 to -0.03; p = .0417).

**Figure 2:**
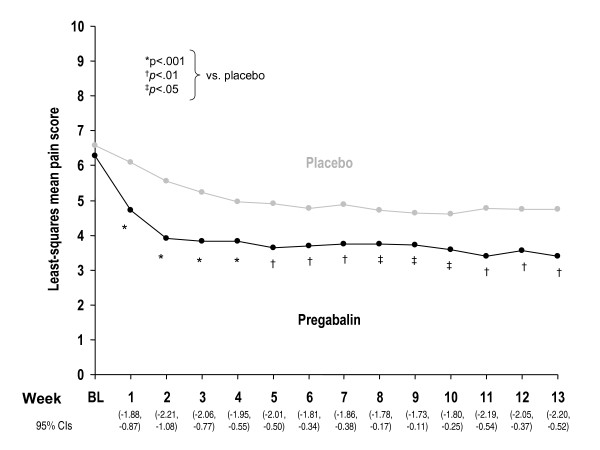
Weekly least-squares mean pain scores.

Supplemental analyses of the primary efficacy parameter included weekly mean pain scores and proportion of responders. Improvement in the pregabalin group was evident at week 1 (p <.0001), the first timepoint evaluated, and this improvement was maintained at every weekly timepoint through week 13 (Figure 2; p ≤.03). At endpoint, a greater proportion of patients in the pregabalin group (49%) met the responder criterion (≥50% reduction in mean pain score from baseline to endpoint) than did those in the placebo group (23%; p <.001).

### Secondary efficacy parameters

Several secondary efficacy parameters were evaluated, including sleep interference, the SF-MPQ, and the CGIC and PGIC. Pregabalin was associated with greater improvement in mean sleep interference scores compared with placebo at endpoint (2.64 vs. 3.72, treatment difference, -1.08; 95% CI, -1.75 to -0.41; p = .0019). This significant improvement was first observed at week 1 and was maintained at every weekly timepoint except for weeks 9 and 10.

### SF-MPQ

The SF-MPQ was administered at screening, randomization, and weeks 1, 5, 9, and 13, and was analyzed separately at each week as well as at endpoint (last observation). On the SF-MPQ sensory, affective, and total scores, numerical comparisons consistently favored pregabalin over placebo. Significant treatment differences were seen at weeks 1 (-5.05; 95% CI, -6.82 to -3.29; p <.0001), 5 (-2.93; 95% CI, -5.04 to -0.82; p = .0068), and 9 (-2.62; 95% CI, -5.20 to -0.04; p = .0464) in the sensory score. On the affective score, a significant treatment difference was seen at week 1 (-1.12; 95% CI, -1.86 to -0.37; p = .0036). For the total score, significant treatment differences from placebo were observed at weeks 1 (-6.13; 95% CI, -8.45 to -3.81; p <.0001) and 5 (-3.43; 95% CI, -6.23 to -0.64; p = .0163).

The SF-MPQ VAS yielded results consistent with those reported above for mean pain score as recorded on the 11-point NRS in patients' daily pain diaries. Pregabalin was significantly better than placebo at each timepoint measured, with the following treatment differences: week 1 = -16.61 (95% CI, -22.85 to -10.36; p <.0001); week 5 = -13.94 (95% CI, -21.98 to -5.90; p = .0008); week 9 = -14.43 (95% CI, -23.09 to -5.76; p = .0013); week 13 = -12.40 (95% CI, -22.55 to -2.25; p = .0173); endpoint = -11.06 (95% CI, -18.89 to -3.22; p = .0060).

For the PPI, treatment differences from placebo, favoring pregabalin, were seen at week 1 (-0.68; 95% CI, -0.97 to -0.39; p <.0001), week 5 (-0.39; 95% CI, -0.74 to -0.04; p = .0294), and at endpoint (-0.34; 95% CI, -0.65 to -0.03; p = .0311).

**Figure 3:**
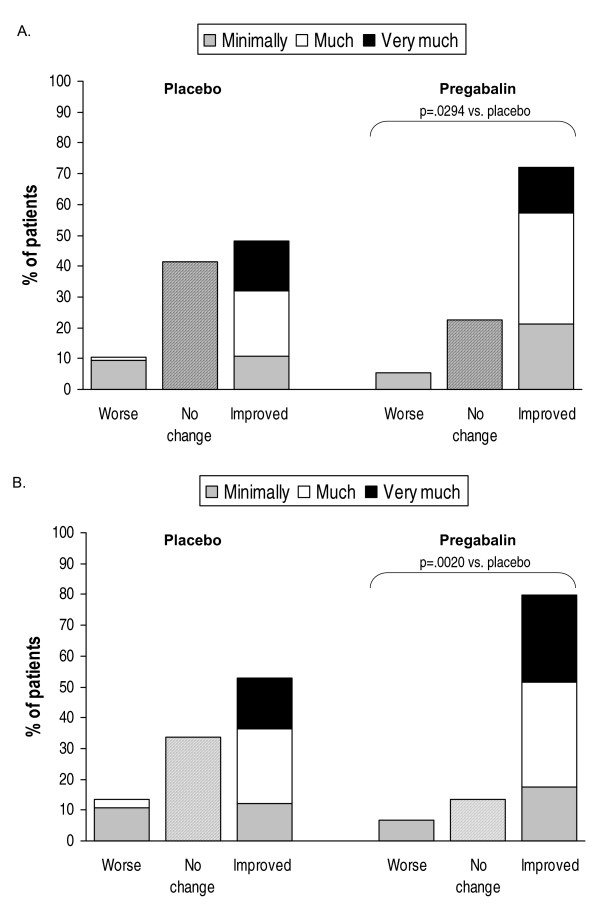
CGIC (A) and PGIC (B) scores.

Global improvement was evaluated with the CGIC and PGIC. On both the clinician-rated and the patient-rated instruments, there was a difference in response favoring pregabalin compared with placebo (CGIC, p = .0294; PGIC, p = .0020) (Figures 3A and 3B).

### Nerve conduction parameters

**Figure 4:**
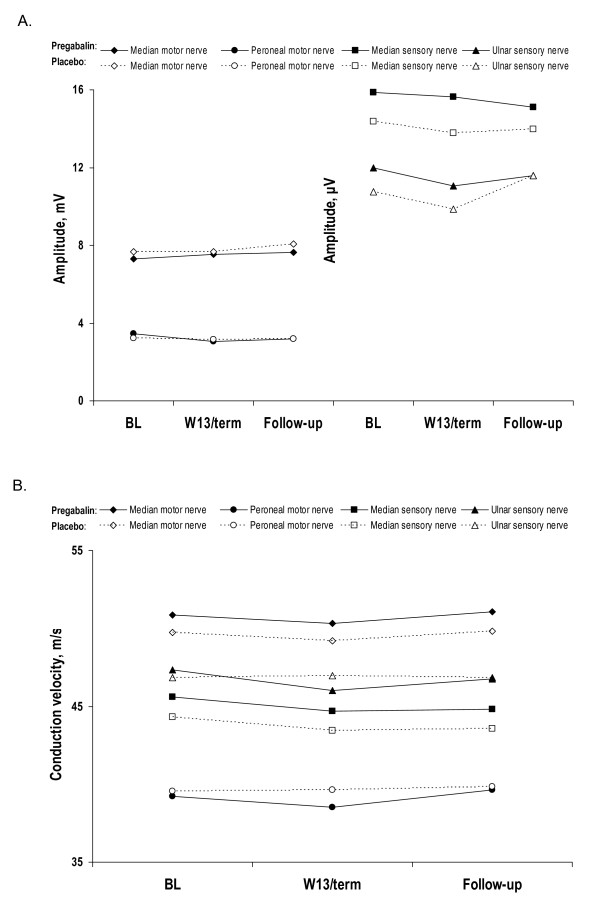
Mean nerve amplitude (A) and nerve conduction (B) in patients treated with 600 mg/d  pregabalin or placebo.

Over 13 weeks of treatment with pregabalin, patients showed no clinically significant changes in nerve conduction measurements (Figures 4A and 4B).

**Figure 5:**
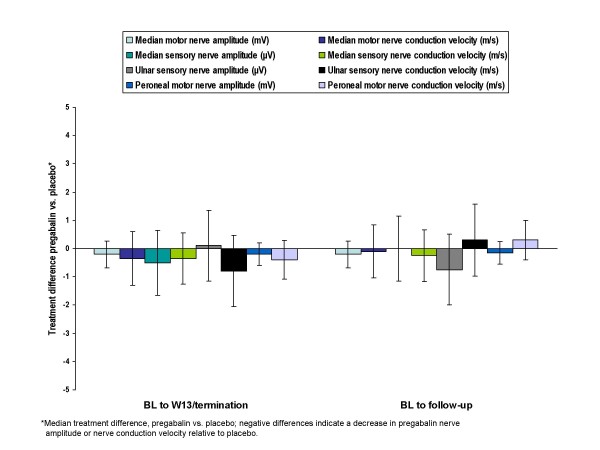
Median differences in nerve conduction parameters between treatment groups: pregabalin 600 mg/d vs. placebo.

Median differences in NC velocity between pregabalin patients and placebo patients in change from baseline to endpoint and change from baseline to follow-up ranged from -0.8 to 0.3 m/s (Figure 5). For each of the 4 nerves assessed, the effect of pregabalin on nerve amplitude and velocity was not significantly different from placebo. Further, none of the changes in NC velocity approached clinical significance, which has been defined, based on a comparative change in the neurologic examination, as a value ranging from 2.2 to 2.9 m/s [32]. More recent discussions with the FDA have placed the boundary of "clinical significance," for the purpose of calculating statistical power, as a change in velocity ranging from 1.2–1.5 m/s (Joseph C. Arezzo, personal communication). The median changes in velocity across all nerves in the present study were substantially less for the pregabalin group than even these more liberal definitions of clinical significance. In summary, there were no statistically significant or clinically meaningful deficits in NC measures associated with pregabalin at a dose clearly efficacious in treating pain (i.e., 600 mg/day).

During the course of the study, the ulnar sensory nerve showed the largest change in median difference between treatment groups. A -0.8 m/s median difference (95% CI, -2.10 to 0.40) in ulnar sensory nerve conduction velocity was observed between treatment groups from baseline to endpoint, with a 0.3 m/s median difference (95% CI, -0.90 to 1.65) from baseline to follow-up. When compared with baseline rather than with placebo, there was minimal actual change in the pregabalin group (i.e., median of -0.35 m/s to endpoint and 0.00 to follow-up).

Although the median changes from baseline were small and non significant, the direction of change in NC measures favored the placebo group in all but 1 measure (ulnar sensory amplitude). Because baseline values were higher for the pregabalin group than for the placebo group in most cases, this could (at least partially) reflect regression to the mean. Differences in scores between the two study groups were often small in absolute value (e.g., a fraction of a μV for sensory amplitude or <0.5 m/s for motor velocities) and were relatively small compared with the observed variance.

### Safety and tolerability

Pregabalin was generally well tolerated. The percentage of patients with ≥1 AEs was similar in the pregabalin (84%) and placebo (78%) groups. There were more associated AEs among the pregabalin group than the placebo group (72% vs. 51%), and incidence of the most frequent associated AEs was higher in the pregabalin than placebo groups: peripheral edema (37%, 32%), dizziness (33%, 6%), weight gain (15%, 1%), and somnolence (13%, 6%) (Table 2). The maximum intensity of most AEs was mild or moderate in both groups, and the percentage of patients with severe AEs was similar in both groups (pregabalin, 10%; placebo, 8%). No patient died during the study. Four patients in the pregabalin group had serious AEs, and one withdrew from the study, whereas in the placebo group, eight patients had serious AEs, and six withdrew from the study. None of these serious AEs were considered associated with treatment, and all patients who experienced serious AEs recovered from the events.

**Table 2:** Most common adverse events considered associated with treatment occurring in ≥3% of either treatment group*

	Placebo (n = 85) Incidence, n (%)	Pregabalin (n = 82) Incidence, n (%)
Peripheral edema	27 (31.8)	30 (36.6)
Dizziness	5 (5.9)	27 (32.9)
Weight gain	1 (1.2)	12 (14.6)
Somnolence	5 (5.9)	11 (13.4)
Asthenia	1 (1.2)	8 (9.8)
Ataxia	0	4 (4.9)
Dry mouth	1 (1.2)	4 (4.9)
Abdomen enlarged	4 (4.7)	3 (3.7)
Edema	0	3 (3.7)
Euphoria	0	3 (3.7)
Thinking abnormal	0	3 (3.7)

*Associated AEs are those considered by the investigator as possibly, probably, or definitely related to study drug and those with insufficient information to determine a relationship.

Rates of discontinuations due to AEs were similar among the pregabalin (17% [14/82]) and placebo (12% [10/85]) groups; however, a greater proportion of AEs leading to discontinuation in the pregabalin group were considered associated with study drug (16% vs. 2%). In the pregabalin group, no single AE accounted for more than five patient discontinuations: peripheral edema (5, 6.1%) and dizziness and somnolence (3, 3.7% for each) were the most commonly cited AEs leading to discontinuation (for each patient, more than one AE could have been cited as a cause of discontinuation). Among study completers, median duration of any AE was similar for patients in the pregabalin (23.5 days) and placebo (29.0 days) groups, though for frequently occurring AEs (peripheral edema, dizziness, and somnolence), the median duration was longer in the pregabalin than in the placebo group.

Weight gain was spontaneously reported as an AE by 18% of pregabalin-treated patients and 4% of placebo patients. The AE was generally considered associated with treatment. Two pregabalin-treated patients withdrew due to weight gain as an AE, but no case of weight gain was reported as an SAE. The percentage of patients with a ≥7% weight gain was higher in the pregabalin group (11%) than in the placebo group (2%).

Neurologic and physical examinations revealed no meaningful differences between patients treated with pregabalin and those receiving placebo. Overall, there were no clinically meaningful changes in laboratory values over the course of the study. Further, diabetes control appeared to be unaffected by study medication. There was a median change of 0 in glycosylated hemoglobin (HbA_1c_) for patients in both the placebo (n = 70) and pregabalin (n = 71) treatment groups (mean change was 0.0029% for placebo patients and -0.07% for pregabalin patients).

## Discussion

At a dosage of 600 mg/d (given 300 mg BID) – representing the upper end of its dosing range – pregabalin showed robust efficacy for the treatment of painful DPN and pain-associated sleep interference, and it was generally well tolerated. These efficacy and safety findings are consistent with those reported in the literature from previous randomized, controlled trials of pregabalin 150 to 600 mg/d administered TID or BID as treatment of painful DPN [9-12,16]. In these earlier studies, significant improvement in pain scores was seen by week 1 [9,10,12] or 2 [9-11,16]; all studies showed significant improvement in sleep interference scores. In the current study, significant differences from placebo in pain and sleep interference were observed at week 1, and these significant improvements were maintained continuously through endpoint for pain scores and at every timepoint, except weeks 9 and 10, for sleep interference.

Results from this 13-week, randomized, double-blind study also demonstrated that pregabalin had no statistically significant or clinically meaningful effect on nerve conduction when dosed at 600 mg/d. Median differences between treatment groups in change from baseline to endpoint and change from baseline to follow-up for all four nerves assessed ranged from -0.8 to 0.3 m/s, with 95% CIs for median differences in amplitude and velocity from baseline to endpoint or to follow-up including 0 in each case. Measure of maximal nerve conduction velocity exclusively reflects changes in large diameter myelinated axons [24], while the pain associated with DPN principally reflects dysfunction in small diameter or unmylinated axons (ie., C-fibers). The NC measures in the present study were included as a safety measure to evaluate the possibility that pregabalin relieves neuropathic pain by causing a broad-based peripheral neuropathy. The findings from the current study demonstrate that pregabalin does not achieve its therapeutic effect of relieving neuropathic pain by damaging or otherwise adversely affecting nerves or nerve function.

Patients responded well to pregabalin; more than twice as many pregabalin (49%) as placebo patients (23%) realized ≥50% improvement in their pain. This finding is consistent with, and the proportion is numerically greater than, responder rates from previous trials of pregabalin that included a fixed-dosage group receiving 600 mg/d, in which proportions of responders ranged from 39% to 48% [10,11,16]. On both clinician- and patient-rated global impressions of improvement, the pregabalin group scored significantly better than the placebo group.

The safety/tolerability profile in this study was generally consistent with previous studies. Most AEs were mild or moderate, with a low incidence of severe AEs and of serious AEs. The overall discontinuation rate and the discontinuation rate due to AEs in the pregabalin treatment group (i.e., 600 mg/day) were within the range of those observed in previous DPN studies at a fixed dose of 600 mg/day [10-12,16]. As in previous trials of pregabalin, dizziness, somnolence, peripheral edema, and weight gain were among the most frequently reported AEs, and they were typically of limited duration. The major difference in AE profile between this and previous trials was that peripheral edema was reported at a higher rate, a finding that we attribute to the difference in procedure for assessment of this AE in this trial. Previous investigations have shown that the frequency of peripheral edema was higher in patients taking pregabalin with a thiazolidinedione antidiabetic agent and did not appear to be attributable to other factors [33].

In previous fixed-dosage studies of pregabalin for painful DPN that included a 600-mg/d treatment group, the incidence of peripheral edema for the 600 mg/d pregabalin group ranged from 10% to 17% and for the placebo group from 2% to 5% [10,11,16]. Of note, the protocol for the current trial required investigators to check for peripheral edema at each visit and to assess whether to report any increase in peripheral edema as an AE. Investigators were not required to report observed edema as an AE if they did not feel it met reporting criteria. This frequent, regular assessment specifically for peripheral edema contributed to the possible ascertainment bias that may help explain the comparatively higher incidence – among both pregabalin and placebo groups – observed in this trial.

This trial is an important addition to the pregabalin and neuropathic pain literature. The robust efficacy demonstrated by pregabalin, together with the lack of effect on nerve conduction, confirm that pregabalin's therapeutic effect on neuropathic pain is not achieved by altering nerve conduction. This finding further elucidates the analgesic activity of pregabalin while substantiating previous findings surrounding the safety of pregabalin as treatment of painful DPN. Additionally, the AE profile emerging from this trial, along with the rapid onset of robust efficacy associated with pregabalin, supports the general tolerability of a more convenient BID dosing schedule – as opposed to a TID dosing schedule – in painful DPN.

## Conclusion

Pregabalin 600 mg/d (300 mg BID) effectively reduced pain, was well tolerated, and had no statistically significant or clinically meaningful effect on NC in patients with painful DPN.

## Competing interests

Joseph C. Arezzo, PhD, served as a consultant for Pfizer, Inc. and received research support from Pfizer, Inc. Julio Rosenstock, MD, served as consultant for Pfizer, Inc. and received research support from Pfizer, Inc for other research not reported in this article. Linda LaMoreaux, MPH was a full-time employee of Pfizer, Inc. during the conduct of the study, and Lynne Pauer, MS is a full-time employee of Pfizer, Inc. Pregabalin is marketed by Pfizer Inc under the brand name Lyrica^®^.

## Authors' contributions

JCA provided expertise regarding nerve conduction for the study design, data analysis, and results interpretation, and was the central reader for nerve conduction assessments. JR contributed to the study design and was a study investigator. LL was lead statistician. LP was the clinical scientist.

## Pre-publication history

The pre-publication history for this paper can be accessed here:


